# A Stretchable Pillararene-Containing Supramolecular Polymeric Material with Self-Healing Property

**DOI:** 10.3390/molecules26082191

**Published:** 2021-04-10

**Authors:** Meng Zhao, Changjun Li, Xiaotao Shan, Huijing Han, Qiuhua Zhao, Meiran Xie, Jianzhuang Chen, Xiaojuan Liao

**Affiliations:** 1 School of Chemistry and Molecular Engineering, East China Normal University, Shanghai 200241, China; 51184300134@stu.ecnu.edu.cn (M.Z.); LCJ0027@163.com (C.L.); sxt1369130195@163.com (X.S.); hjhan@chem.ecnu.edu.cn (H.H.); qhzhao@chem.ecnu.edu.cn (Q.Z.); mrxie@chem.ecnu.edu.cn (M.X.); 2 School of Materials Science and Engineering, East China University of Science and Technology, Shanghai 200237, China

**Keywords:** pillararene, host-guest chemistry, supramolecular chemistry, supramolecular polymer

## Abstract

Constructing polymeric materials with stretchable and self-healing properties arise increasing interest in the field of tissue engineering, wearable electronics and soft actuators. Herein, a new type of supramolecular cross-linker was constructed through host-guest interaction between pillar[5]arene functionalized acrylate and pyridinium functionalized acrylate, which could form supramolecular polymeric material via photo-polymerization of n-butyl acrylate (BA). Such material exhibited excellent tensile properties, with maximum tensile strength of 3.4 MPa and strain of 3000%, respectively. Moreover, this material can effectively dissipate energy with the energy absorption efficiency of 93%, which could be applied in the field of energy absorbing materials. In addition, the material showed self-healing property after cut and responded to competitive guest.

## 1. Introduction

Polymeric materials with stretchable property have got plenty of attention due to their widespread applications in the fields of tissue engineering, wearable electronics and soft actuators [1,2,3,4,5,6,7]. It is well known that there is always a trade-off between strength and toughness for the stretchable polymeric materials [8,9,10]. In general, strong interactions and rigid structures, such as covalent bonded gels, often provide higher tensile strength but with very low strain at break and slow self-healing, while weaker interactions lead to faster self-healing, but soft and viscoelastic materials [11,12,13,14]. For example, Huang reported a polymer gel based on the formation of acylhydrazone bonds [15]. The tensile breaking stress reached 1.2–2.7 MPa after solvent exchange from DMSO to water. However, the gel did not show self-healing behavior and the maximum strain at break was 40%, which limited their application. Therefore, it is still a challenge to explore a facile method to obtain polymeric materials with excellent strength and toughness as well as self-healing property.

Scientists have made great efforts to solve this problem by combining covalent bonded polymer with non-covalent interactions, including H-bond and coordination bond [16,17,18]. Among these various non-covalent interactions, host-guest chemistry, the central of supramolecular chemistry, has attracted tremendous attention, since it endows the system with reversible, tunable and dynamic properties [19,20,21,22,23,24,25,26]. For example, Suzuki and Takata reported the formation of tough films by evaporation of water from dispersions of elastomer microspheres crosslinked with rotaxane supramolecules, which contained crown ether [27]. Guo and colleagues prepared ultraductile, notch and stab resistant supramolecular hydrogels by copolymerization of acrylamide and adamantane monomer in the presence of cyclodextrin-functionalized polymer [28]. As the pioneer in this field, Harada and colleagues prepared flexible and tough elastomers with self-healing abilities by using cyclodextrin derivatives [29]. Hence, host–guest interactions could make great contributions in preparation of polymeric materials with good mechanical and self-healing properties.

On the other hand, pillararenes, as a new fascinating family of macrocyclic host molecules, have attracted increasing attention due to their novel symmetric structures and extensive applications in the construction of novel supramolecular architectures [30,31,32,33,34,35,36]. For instance, Ogoshi succeeded in molecular weight fractionation by the confinement of single polymer chains of poly(ethylene oxide) in the one-dimensional channels of crystalline pillar[5]arene [37]. Huang constructed adaptive photosensitizers based on a host-guest complex containing a water-soluble pillar[5]arene and an AIEgen photosensitizer [38]. However, few supramolecular polymeric materials based on pillararene were reported [39,40,41], of which the mechanical performance was not good enough and need to be improved.

**Scheme 1:**
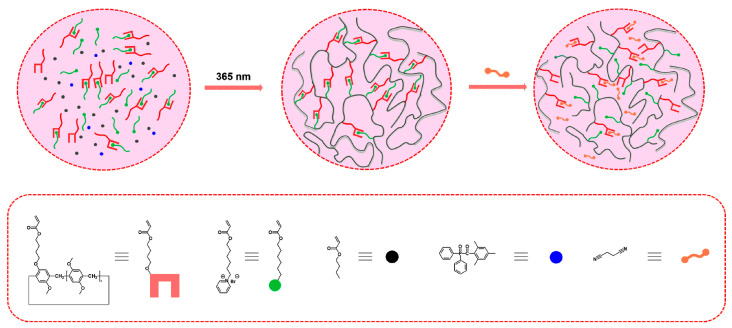
Schematic illustration of preparation of stretchable supramolecular polymeric material with self-healing property.

Herein, we successfully prepared a new type of supramolecular polymeric material with excellent stretchable and self-healing properties. Firstly, a bifunctional cross-linker of host-guest supramolecule (HGS) was constructed by mixing pillar[5]arene functionalized acrylate (AAP5A) with pyridinium functionalized acrylate (G) in acetone. Then, *n*-butyl acrylate (BA) was added and further transformed into a cross-linked supramolecular polymeric material (SP) by free radical polymerization under UV light (Scheme 1). Such material exhibited excellent tensile properties, with maximum tensile strength of 3.4 MPa and strain of 3000%, respectively. Moreover, this material can effectively dissipate energy with the energy absorption efficiency of 93%, which could be applied in the field of energy absorbing materials. In addition, the material showed self-healing property after cut and responded to competitive guest.

## 2. Results and Discussion

**Scheme 2:**
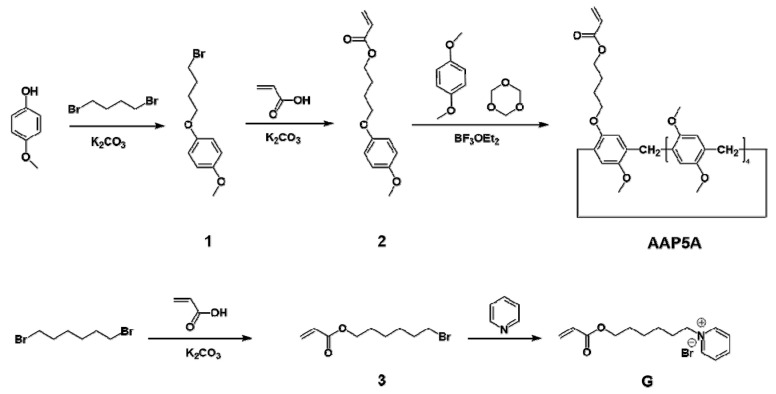
Synthetic routes of AAP5A and G.

**Figure 1:**
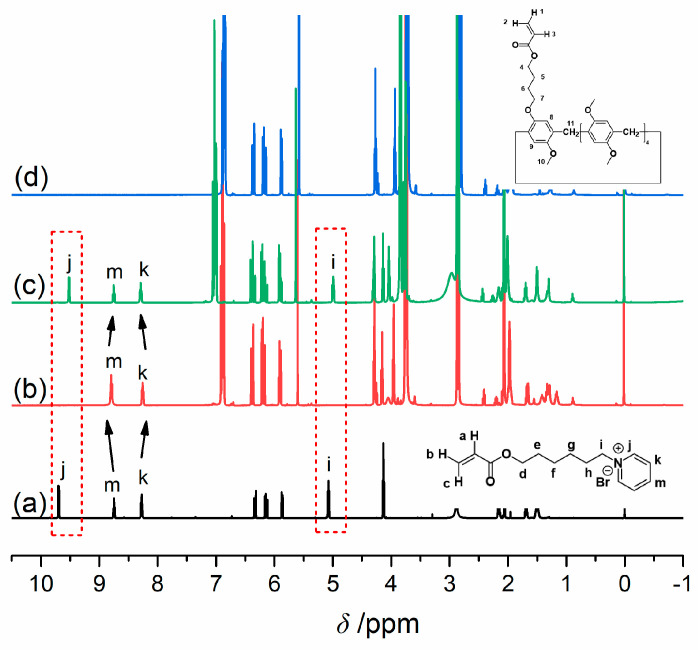
^1^H-NMR spectra (500 MHz, acetone-d6, 298 K) of 4 mM G (**a**), 4 mM G + 8 mM AAP5A (**b**), 4 mM G + 8 mM AAP5A +20 mM G1(**c**) and 8 mM AAP5A (**d**).

To construct the supramolecular crosslinker, pillar[5]arene functionalized acrylate (AAP5A) and pyridinium functionalized acrylate (G) were synthesized and characterized. (Scheme 2, Figures S1–S11, Supplementary Materials). The host-guest interaction between AAP5A and G was firstly studied by ^1^H-NMR (Figure 1). Curves a and d refer to the solution of G and AAP5A, respectively. The addition of AAP5A to G solution leads to remarkable changes (Figure 1, curve b), that is, the signals of protons H_j_ from pyridinium group and H_i_ from methylene group of G broadened severely and the signal of proton H_k_ shifted upfield. According to the literature [42], the complexation induced upfield shift and broadening effect were remarkable when the carbon chain was long. On the other hand, the signal of proton H_m_ shifted downfield, due to the shielding effect of the electron-rich cavities of pillararenes. All of these phenomena revealed that the pyridinium and methylene groups threaded into the cavity of pillararenes similar to the literatures reported [42,43]. Furthermore, 2D NOESY spectrum showed the correlation peaks between H_j−k_ and H_m_ of G and H_10−11_ of AAP5A (Figure S12), indicating that the guest molecule G successfully entered the cavity of AAP5A, which demonstrated the host-guest interaction between AAP5A and G. On the basis of ^1^H-NMR titration experiment (Figure S13) and the nonlinear curve fitting method (Figure S14), the binding constant of AAP5A with G was calculated to be 156 ± 41 mol·L^−1^. According to the literature reported, the driving force of this supramolecular system can be attributed to the combination of hydrophobic and cation-π interaction between electron-lacking G and electron-rich P5A cavities [44].

**Figure 2:**
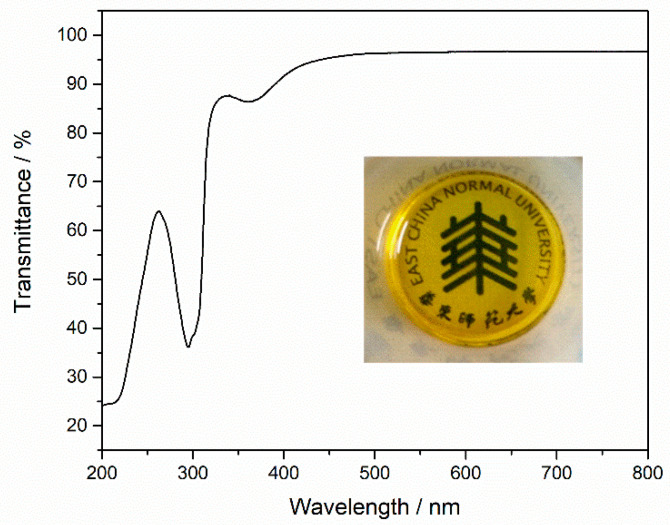
UV-vis transmittance and the picture (inset) of the supramolecular material.

Since the cavity of AAP5A can be threaded by the pyridinium and methylene groups of G, it is possible to construct a supramolecular cross-linker based on the pillararene-pyridinium group recognition motif. Next, polymerization of the monomer *n*-butyl acrylate (BA) was performed in acetone, using 2,4,6–trimethylbenzoyldiphenylphosphine oxide (TPO) as the photo initiator and the host-guest supramolecule (HGS) as supramolecular crosslinker and a supramolecular polymeric material with optical transparency (Figure 2, inset) was obtained. Since the polymerizable groups were the same in BA, AAP5A and G, their reaction activities were similar, which made the component proportion in the network facile to be adjusted by varying the feed ratio. As shown in Figure 2, the transmittance reached 96% in the wavelength range of 500–800 nm. Concerned the 3D network of supramolecular materials, FT-IR characterization was facile to take insight into this polymerization process. As shown in Figure S15 (Supplementary Materials), curves a and b referred to the signals of AAP5A and G, respectively. Mixing AAP5A with G lead to some changes. As shown in Figure S15, curve c, the stretching vibration peak of C-H (3037 cm^−1^) and the aromatic group (1581 cm^−1^) as well as the out-of-plane bending vibration absorption peak of trisubstituted C-H (894 cm^−1^) from the pyridinium group of G disappeared, which was caused by the host-guest interaction between AAP5A and G. After polymerization, as shown in Figure S15, curve d, the out-of-plane bending vibration absorption peaks of benzene group (880, 853 cm^−1^) on the pillararene did not move significantly, while the out-of-plane bending vibration absorption peak of C-H (810 cm^−1^) from C=C disappeared, indicating that the raw material basically reacted completely in the process of photo polymerization. In addition, the stretching vibration absorption peak of the carbonyl group upshifted from 1718 to 1728 cm^−1^, due to the disappearance of the double bond, which destroyed the conjugated system of the double bond with the carbonyl group. All the phenomena revealed the successful polymerization of double bond and the formation of polymeric material.

**Figure 3:**
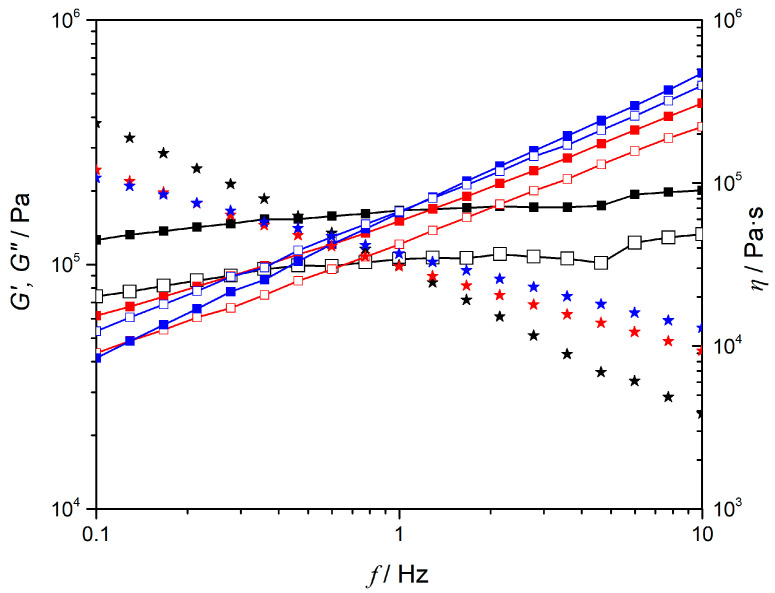
Rheology results of the supramolecular material at 4 °C. (SP-1 in black line, SP-2 in red line, SP-3 in blue line; storage modulus G′ (■), loss modulus G″ (□), complex viscosity η (★)).

The density of crosslinker may have influence on the mechanical properties [45,46,47]. In order to investigate it further, three supramolecular polymeric materials (named SP-1, SP-2 and SP-3, respectively) with different content of host-guest supramolecule HGS (9, 7 and 5%) were prepared. The feed ratio was shown in Table S1 in detail. In the rheological test (Figure 3), the storage modulus G′ of the two samples SP-1 and SP-2 were both greater than the corresponding loss modulus G″ and there was no crossover within the experimental test range, indicating that these two samples were stable. As compared, the storage modulus G′ and loss modulus G″ of sample SP-3 crossed at the frequency of 1.00 Hz, showing its gelation ability becomes weak. In addition, the rheological test showed that the G′ and G″ of SP-1 hardly changed as the frequency varied and G′ >> G″ compared with the other two samples, revealing SP-1 is most stable among these samples. Therefore, with increasing of the content of HGS, the crosslinking density increased, leading to the enhancement of strength of these supramolecular polymeric materials. Previously, we reported a supramolecular gel with G′ of only 2 KPa by mixing pillararene-containing copolymers with a bis(pyridinium) dication guest [40]. Interestingly, as compared, the G′ of SP-1 was higher (1.5 × 10^5^ Pa) by two orders of magnitude, indicating that mixing host-guest first and then doing the polymerization can effectively improve the mechanical stability of the supramolecular polymeric materials.

To compare this supramolecular polymeric material with traditional network crossslinked by covalent crosslinker, some polymeric materials (samples 1–5) with different content of crosslinker diethylene glycol diacrylate (DEGDA) were prepared (Table S1). To our surprise, the covalent cross-linked polymeric materials were very brittle and could not form a fixed shape after photo polymerization (Figure S16, Supplementary Materials). Therefore, we failed to do the tensile tests. On the other hand, samples prepared with the ratio of HGS to BA with 1:10, 1:15, 1:20 were tough enough to form supramolecular gels, but samples with the ratio of HGS to BA with 1:3, 1:5 could not (Table S1). According to the literature reported [39], this may be caused by the high content of pillararenes, which brought about steric hindrance and prevented the polymerization.

**Figure 4:**
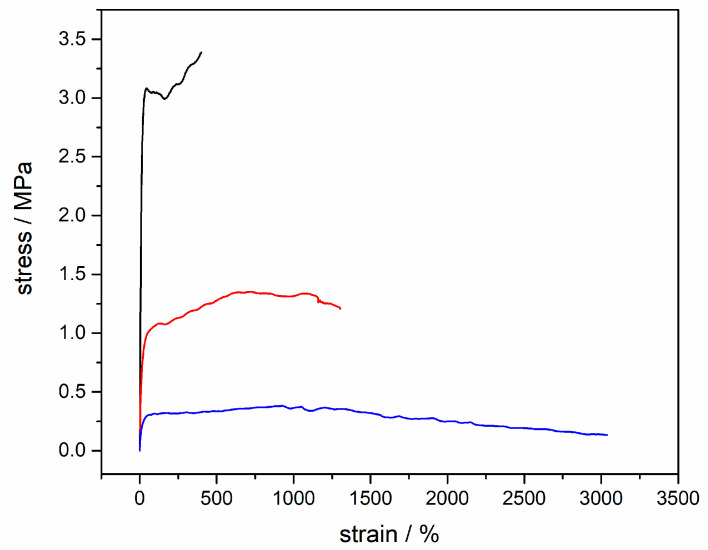
The stress−strain curves of SP-1 (black curve), SP-2 (red curve) and SP-3 (blue curve).

With the increasing of HGS content, the tensile strength of supramolecular polymeric materials increased significantly from 0.4 MPa to 3.4 MPa (Figure 4, Table S2), which was in good agreement with the rheological results. This was mainly caused by the rigid structure of pillararenes which formed the “hard segment” in the network and provided strong mechanical property. On the other hand, while the non-covalent cross-linking density inside the polymeric materials increased, the modulus of the materials increased accordingly; hence, the tensile strength of the materials increased significantly. Learning from the literatures [48], we speculated that small clusters of supramolecular crosslinkers with local high concentration may exist after polymerization, due to the π-π conjugation effect arising from pillararenes. Therefore, wide-angle X-ray scattering (XRD) measurements of three samples were carried out. As shown in Figure S17, all these samples showed a characteristic peak at 2*θ* = 22.5°, which was attributed to the ordered accumulation of pillararenes arising from the π-π interaction and lead to the high tensile strength of the material. According to Bragg’s Law (*λ* = 2*d*sinθ), the corresponding spacing distance *d* was 0.395 nm. Compared with the regular structure formed by poly(ethylene glycol) (PEG) and α-cyclodextrin (2*θ* = 19.8°), the spacing distance of this pillararene-based regular structure was smaller, revealing that the supramolecular polymeric material was more transparent than those prepared from PEG and cyclodextrin, which is usually opaque [49].

**Figure 5:**
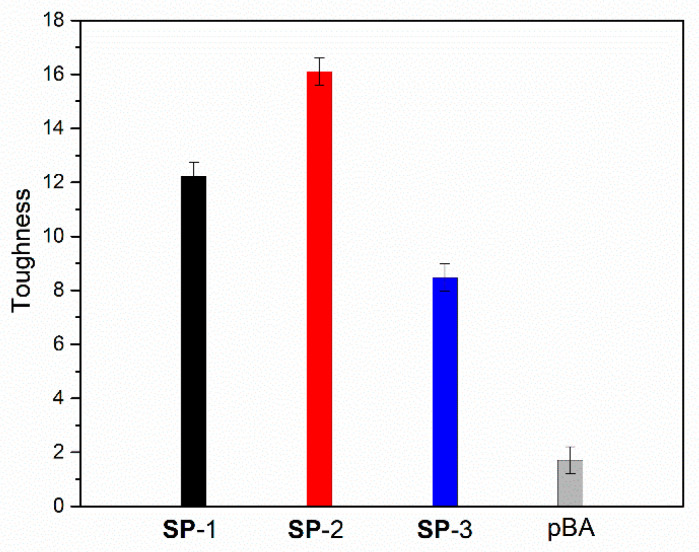
The toughness of SP-1 (black), SP-2 (red) and SP-3 (blue) and pBA (grey).

The polymeric materials prepared in this way were very flexible as the elongations at break varied from 400 to 3000% and the toughness of the SP-2 could reach 16.11 MJ·m^−3^ (Figure 5), which was much greater than the polybutyl acrylate (pBA) (1.71 MJ·m^−3^). Moreover, necking appeared in the latter stage of stretching, which was a typical ductile fracture behavior [50,51]. It was speculated that the butyl acrylate flexible group constituted the “soft segment” of the network, which effectively relieved the rigidity of pillararenes, acting as the “hard segment”. To demonstrate this speculation, differential scanning calorimetry (DSC) characterizations were conducted. As shown in Figure S18, the glass transition temperature (*T_g_*) of the supramolecular material increased with the increasement of supramolecular crosslinker and the highest *T_g_* was below the room temperature, indicating that the supramolecular crosslinked materials were flexible. Therefore, the rigid pillararenes and soft polybutyl acrylate chains, as well as the dynamic noncovalent bonds played important roles in enhancing the toughness of this supramolecular polymeric materials.

**Figure 6:**
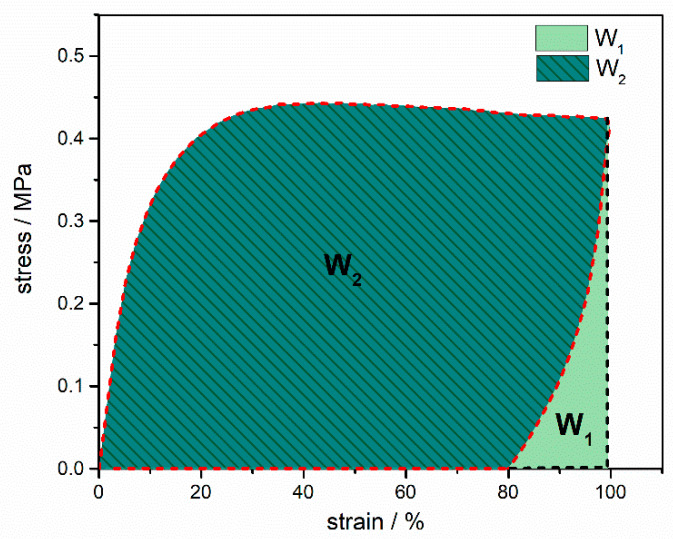
The energy absorbing properties of SP-1.

It is well known that the mechanical properties of materials usually involve a trade-off between modulus and elongation. In general, strong interactions often result in higher tensile strength with the loss of toughness, which makes the material brittle and hard. The reversible dynamic non-covalent bond can achieve higher toughness and self-healing properties of the material, as well as dissipate energy effectively, so it can be used as a candidate for energy-absorbing materials [52]. As shown in Figure 6, there was an obvious hysteresis appeared in the cyclic stress–strain test with a fixed applied strain of 100% and the energy absorption efficiency *ω* was as high as 93%, indicating that the reversible host–guest interaction led toeffective dissipation of energy. When subjected to external forces, the flexible host–guest supramolecules with ability to move can effectively dissipate the external force, which could be used in the field of energy-absorbing materials.

**Figure 7:**
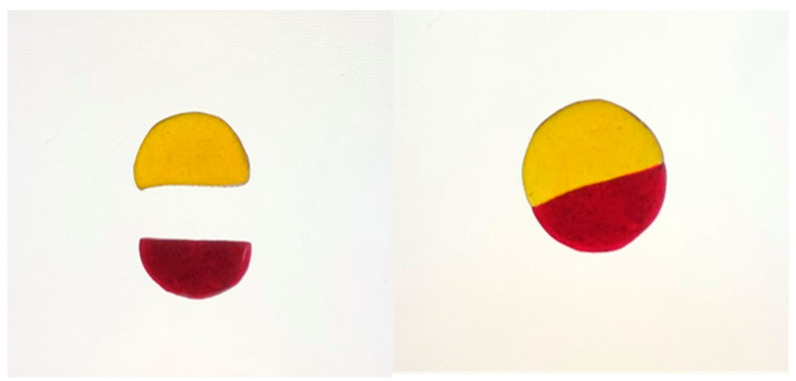
The photos of the two cut samples (**left**) and re-formed one (**right**).

The host-guest complexation is a reversible dynamic non-covalent bond, which may enable the polymeric materials with self-healing property. In order to study the self-healing behavior of this material, two samples of different colors (one sample was stained with rhodamine B for better observation) were cut into two pieces with the same size and then the different samples were put together to make the sections contacted. After 30 min, we found that the two samples were connected as a whole (Figure 7). In contrast, the covalent bond cross-linked samples could not form an integrity after being cut. Moreover, this reformed material could sustain a load of 333 g and still endure a large strain (Figures S19–S20, Supplementary Materials). These results indicated that we have successfully constructed a supramolecular polymeric material with self-healing property.

**Figure 8:**
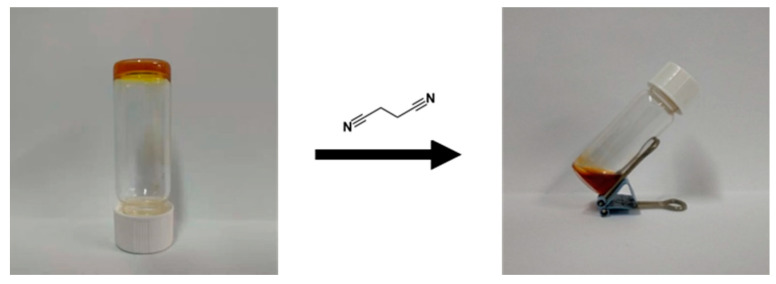
Reversible gel−sol transition of the supramolecular polymeric material.

This supramolecular polymeric material may be responsive to chemical stimulus owing to the dynamic non-covalent bond. According to the literature [53], the strong interaction between butanedinitrile (G1) and pillararene can push the pyridinium group out of the pillararene cavity. Therefore, we choose G1 to investigate the competitive process. First, the complexation behavior of AAP5A, G and G1 was studied by ^1^H-NMR (Figure 1, curve c). After five times equivalent of G1 was added to the mixed solution of AAP5A and G, the signals of the protons H_j_ and H_i_ of G appeared again, the protons signal of H_k_ and H_m_ returned to the initial values, which proved that G1 and AAP5A formed a stronger complex in acetone and could destroy the structure of the supramolecular cross-linker formed by AAP5A and G. Therefore, with the addition of excessive amount of G1, the polymeric material turned to be a flowing sol state (Figure 8), showing the stimulus responsiveness to competitive guest molecules.

## 3. Materials and Methods

1,4-Dibromobutane (99%), 1,6-Dibromohexane (98%), 4-methoxyphenol, acrylic acid, paraformaldehyde (99%) and boron trifluoride etherate (48%) were purchased from Shanghai Titan Scientific Co. (Shanghai, China) and used as received. 1,4-Dimethoxybenzene was purchased from Aladdin Industrial Corporation (Shanghai, China). Pyridine was purchased from Sinopharm Chemical Reagent Co. (Shanghai, China). TPO was purchased from Shanghai Excellent Chemical Co. Ltd. All other solvents were obtained from commercial suppliers and were used directly without further purification. Ultrapure water was used in all relevant experiments.

^1^H-NMR (500 MHz) and ^13^C-NMR (126 MHz) spectra were recorded on a Bruker DPX500 spectrometer (Bruker BioSpin, Switzerland) using tetramethylsilane as an internal standard in CDCl_3_ or acetone-*d_6_*.MALDI-TOF was recorded on an UltrafleXtreme III MALDI-TOF mass spectrometer (Bruker Daltonics, Germany). UV-vis spectra were recorded on a Shimadzu UV-1800 spectrophotometer (Shimadzu, Japan). FT-IR spectroscopy was obtained on a NEXUS 670 FTIR spectrometer (Thermo, USA). The rheological properties were studied by a MARS-3 (Thermo Hakke Instruments, USA). Frequency sweeps from 0.01 to 100 Hz were performed at 0.1% strain under 4 °C on circular samples (20 mm diameter). Uniaxial tensile measurements were performed on a tensile testing machine HY-0508 (Shanghai Hengyi Test Instrument Co., Ltd., China) equipped with a 50 N load cell with a crosshead speed of 30 mm min^−1^ at room temperature. The testing rectangular samples were prepared with the length of 5 mm, width of 5 mm and thickness of 0.8~1 mm. As for the loading-unloading test and the successive loading-unloading tests, the crosshead velocity were both kept at 30 mm min^−1^. The toughness was calculated from the area of stress-strain curves [54]. The energy absorbing efficiency ω were calculated according to the calculate equation in the literature [52]. Small angle and wide angle X-ray scattering were performed on a Smartlab SE (Bruker, Germany). All samples were prepared with thickness of <1 mm. DSC was performed on a Q2000 DSC (TA, USA) in nitrogen atmosphere. All the samples were first heated from 25 to 180 °C at a rate of 50 °C min^−1^ and held at this temperature for 3 min to eliminate the thermal history; then, they were cooled to −90 °C and heated again from −90 to 180 °C at a heating or cooling rate of 10 °C min^−1^.

## 4. Conclusions

In conclusion, we have successfully constructed a new type of host–guest supramolecular crosslinker based on pillar[5]arene functionalized acrylate and pyridinium functionalized acrylate, which can form a supramolecular polymeric material through photo polymerization of *n*-butyl acrylate. Such supramolecular polymeric material exhibited tunable stretchable property by varying the content of the supramolecular cross-linker. In addition, this material showed effective dissipation of energy, which could be applied in the field of energy absorbing materials. Moreover, this material exhibited self-healing property and stimulus response to competitive guest molecules, which were obviously different from covalent bond crosslinked network. This work provides an effective method for preparation of supramolecular polymeric materials with tunable stretchable, self-healing, chemical responsive and energy dissipative properties and offers a new paradigm for preparing multi-functional materials.

## Supplementary Materials

The following are available online, Experimental details, Figures S1–S7: ^1^H-NMR, ^13^C-NMR spectra and MALDI-TOF of **AAP5A**; Figures S8–S11: ^1^H-NMR, ^13^C-NMR spectra of **G**; Figure S12: NOESY spectrum of a mixture of **AAP5A** and **G**; Figures S13–S14: ^1^H-NMR titration experiment and the nonlinear curve fitting method of **AAP5A** and **G**; Figure S15: FT-IR spectra of **AAP5A**, **G**, **HGS**, and **SP**-1; Figure S16: The picture of the covalent cross-linked polymeric material; Table S1: Feeding ratio of polymeric materials; Table S2. Summary of mechanical properties, Table S2: Summary of mechanical properties; Figure S17: Wide angle X-ray scattering of SP; Figure S18: DSC curves of **SP**; Figures S19–S20: Self-healing property of the supramolecular polymeric materials.

## Sample Availability

Samples of the compounds **AAP5A** and **G** are available from the authors.
