# Supplementary material for: A Stretchable Pillararene-Containing Supramolecular Polymeric Material with Self-Healing Property
Source: Molecules. 2021 Apr 10;26(8):2191. doi: 10.3390/molecules26082191 (PMC8070141; doi:10.3390/molecules26082191)
Supplement: Supplementary file 1 [file molecules-26-02191-s001.pdf]

Supporting information

A Stretchable Pillararene-Containing Supramolecular Polymeric Material with Self-Healing Property

Meng Zhao[1], Changjun Li[1], Xiaotao Shan[1], Huijing Han[1], Qiuhua Zhao[1], Meiran Xie[1], Jianzhuang Chen[2,*], Xiaojuan Liao[1,*]

[1] School of Chemistry and Molecular Engineering, East China Normal University, Shanghai, 200241, China

[2] School of Materials Science and Engineering, East China University of Science and Technology, Shanghai 200237, China.

*** Correspondence:** chenjianzhuang@ecust.edu.cn (J.Z.C.);

xjliao@chem.ecnu.edu.cn (X.J.L.).

Table of Contents

1. Synthesis and Characterizations 2

Synthesis of compound 1 2

Synthesis of compound 2 3

Synthesis of pillar[5]arene functionalized acrylate (AAP5A) 4

Synthesis of compound 3 6

Synthesis of pyridinium functionalized acrylate (G) 8

2. Host-guest complexation studies of the supramolecular cross-linking molecule composed by AAP5A and G 10

NMR titration study of the complexation between AAP5A and G 10

Association constant determination for the supramolecular cross-linking molecules composed by AAP5A and G 11

3. Preparation of the supramolecular polymeric material 12

4. Characterizations of the supramolecular polymeric materials 13

5. Self-healing property of the supramolecular polymeric materials 15

References 16

1. Synthesis and Characterizations

Synthesis of compound 1

Compound **1** was synthesized according to the previous procedure.[S1] The ^{1}H NMR spectrum of the compound **1** is shown in **Figure S1**. ^{1}H NMR ($CDCl_3$, 500 MHz, 298K) δ (ppm): 6.83 (s, 4H), 3.95 (t, J = 6.1 Hz, 2H), 3.77 (s, 3H), 3.49 (t, J = 6.7 Hz, 2H), 2.11 – 2.02 (m, 2H), 1.92 (dq, J = 9.6, 6.2 Hz, 2H). The ^{13}C NMR spectrum of the compound **1** is shown in **Figure S2**. ^{13}C NMR (126 MHz, $CDCl_3$, 298K) δ (ppm): 153.86, 153.03, 115.43, 114.68, 77.30, 77.05, 76.79, 67.49, 55.76, 33.54, 29.53, 28.03.

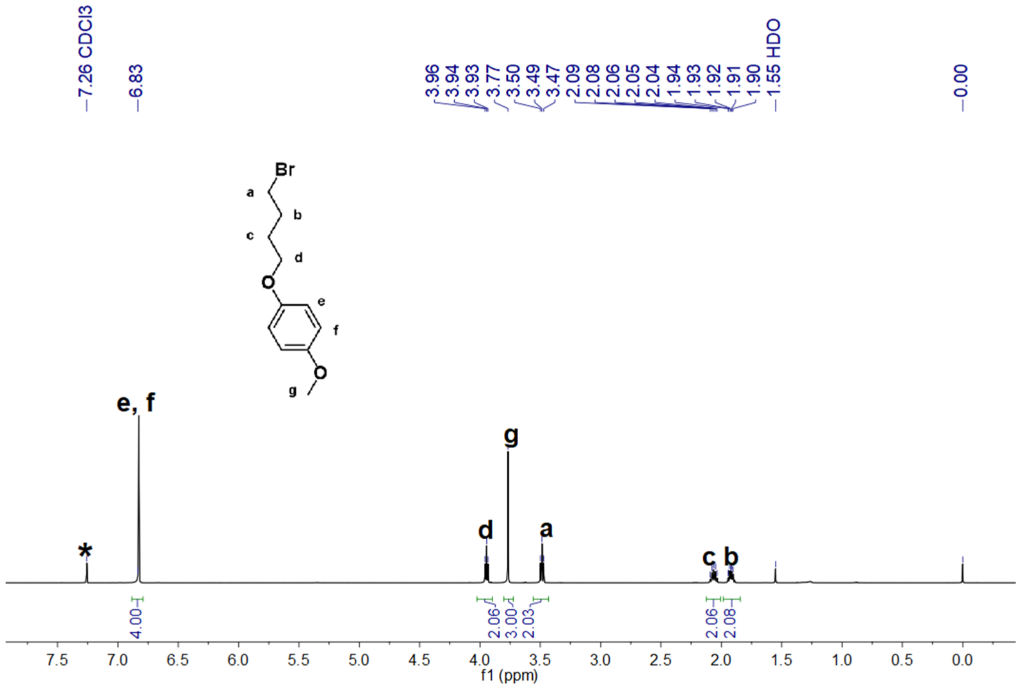

Figure S1. ^{1}H NMR spectrum (500 MHz, $CDCl_3$) of compound **1**.

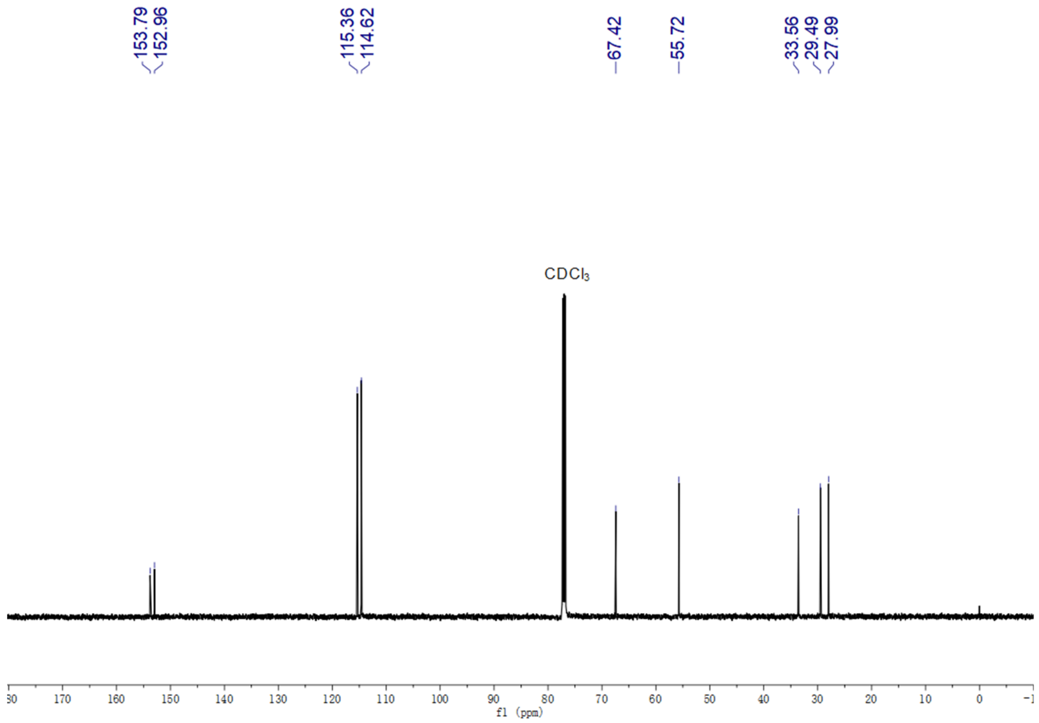

Figure S2. ^{13}C NMR spectrum (126 MHz, $CDCl_3$, 298K) of compound **1**.

Synthesis of compound 2

The compound **1** (5.2 g, 20 mmol), K_2CO_3 (27.6 g, 100 mmol) and KI (5.0 g, 20 mmol) were dissolved in CH_3CN (200 mL). After complete dissolution, acrylic acid (4.3 g, 60 mmol) was added drop by drop, and stirred at 80 °C under N_2 atmosphere for three days. After the reaction, the filtrate was cleaned and filtered by dichloromethane for several times. The filtrate was combined and concentrated. The crude product was further purified by silica gel column chromatography (eluent: petroleum ether/ethyl acetate = 10:1, v/v). The yellowish viscous liquid was obtained of 4.2 g (yield: 83%). The ^{1}H NMR spectrum of compound **2** is shown in **Figure S3**. ^{1}H NMR ($CDCl_3$, 500 MHz, 298K) δ (ppm): 6.83 (s, 4H), 6.40 (dd, J = 17.3, 1.5 Hz, 1H), 6.12 (dd, J = 17.4, 10.4 Hz, 1H), 5.82 (dd, J = 10.4, 1.4 Hz, 1H), 4.26 – 4.21 (m, 2H), 4.01 – 3.91 (m, 2H), 3.77 (s, 3H), 1.93 – 1.81 (m, 4H). The ^{13}C NMR spectrum of compound **2** is shown in **Figure S4**. ^{13}C NMR (126 MHz, $CDCl_3$, 298K) δ (ppm): 166.27, 153.79, 153.06, 130.76, 128.49, 115.42, 114.64, 67.91, 64.24, 55.73, 25.99, 25.43.

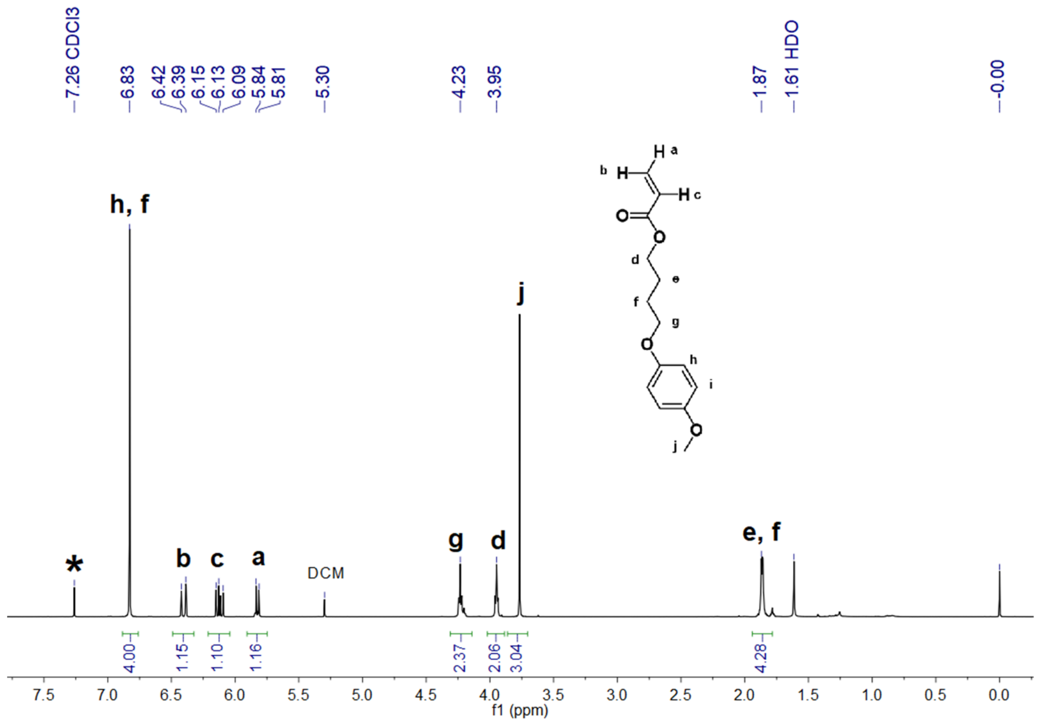

Figure S3. ^{1}H NMR spectrum (500 MHz, $CDCl_3$) of compound **2**.

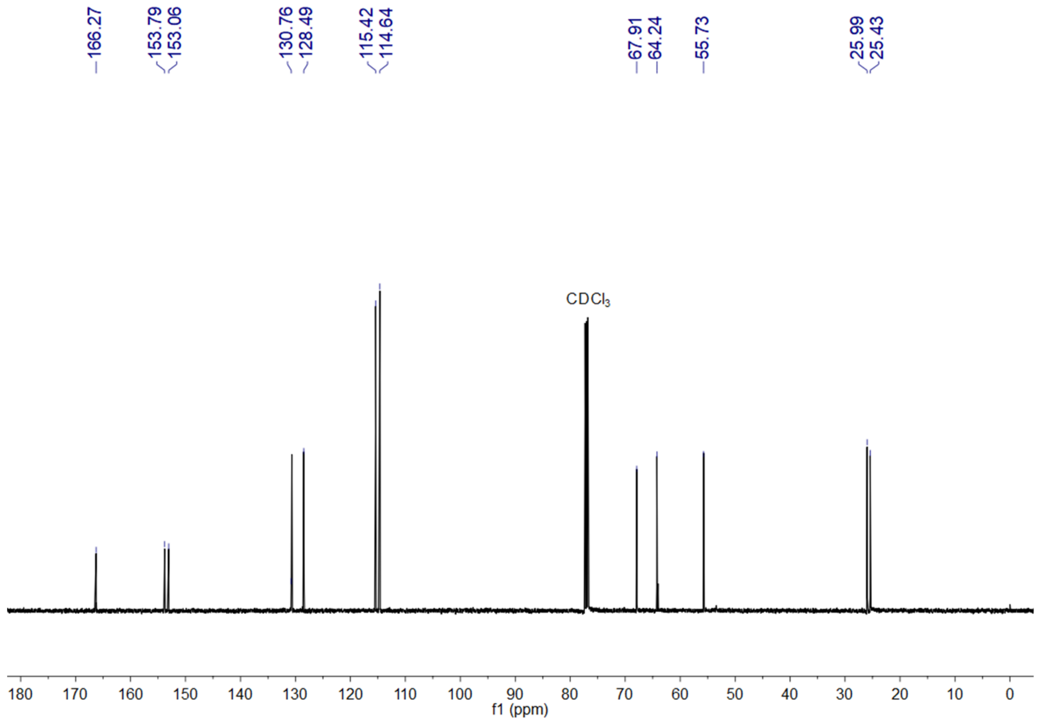

Figure S4. ^{13}C NMR spectrum (126 MHz, $CDCl_3$, 298K) of compound **2**.

Synthesis of pillar[5]arene functionalized acrylate (AAP5A)

The compound **2** (0.3 g, 1 mmol), 1,4-dimethoxybenzene (0.5 g, 4 mmol) and

paraformaldehyde (0.45 g, 5 mmol) were added into the solution of 1,2-dichloroethane (200 mL). After all the raw materials were dissolved, the reaction system was kept at 30°C for a period of time and then boron trifluoride ether (BF_3OEt_2, 1.25 mL, 10 mmol, 48%) was added 5 minutes later, the raw materials were completely reacted and quenched with water. The liquid was separated, extracted and combined with organic phase. The anhydrous Na_2SO_4 was dried, filtered and concentrated to obtain the crude product, which was further purified by silica gel column chromatography (eluent: petroleum ether/dichloromethane = 1:1, v/v). The white solid was obtained of 0.1g (yield: 12%). The ^{1}H NMR spectrum of **AAP5A** is shown in **Figure S5.** ^{1}H NMR ($CDCl_3$, 500 MHz, 298K) δ (ppm): 6.89 – 6.65 (m, 10H), 6.37 (dd, J = 17.4, 1.4 Hz, 1H), 6.11 (d, J = 10.5 Hz, 1H), 5.78 (dd, J = 10.4, 1.4 Hz, 1H), 4.24 (t, J = 6.2 Hz, 2H), 3.87 (t, J = 5.9 Hz, 2H), 3.83 – 3.71 (m, 10H), 3.72 – 3.59 (m, 27H), 1.99 – 1.75 (m, 4H). The ^{13}C NMR spectrum of **AAP5A** is shown in **Figure S6**. ^{13}C NMR (126 MHz, $CDCl_3$, 298K) δ (ppm): 166.23, 150.66, 149.76, 130.73, 128.39, 128.26, 128.17, 114.70, 113.95, 113.89, 113.80, 67.71, 64.29, 55.70, 52.97, 39.38, 29.67, 29.60, 29.54, 29.49, 29.35, 26.42, 25.67. MALDI-TOF of **AAP5A** is shown in **Figure S7**. m/z calcd for $[C_{51}H_{60}NaO_{12}]^+$: 863.010, found: 862.708.

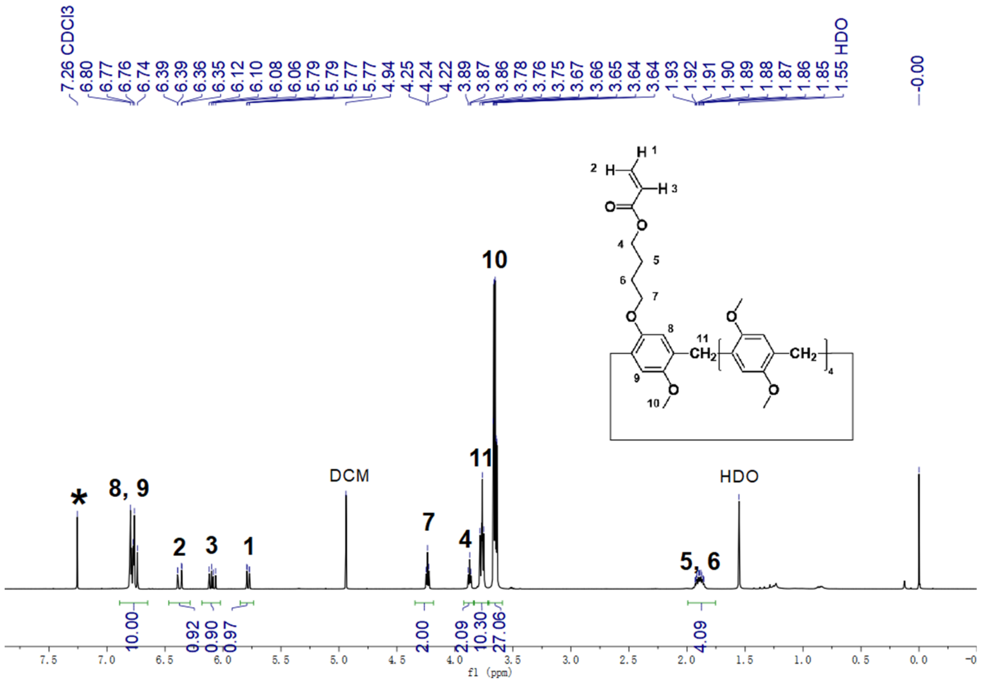

Figure S5. ^{1}H NMR spectrum (500 MHz, $CDCl_3$) of **AAP5A**.

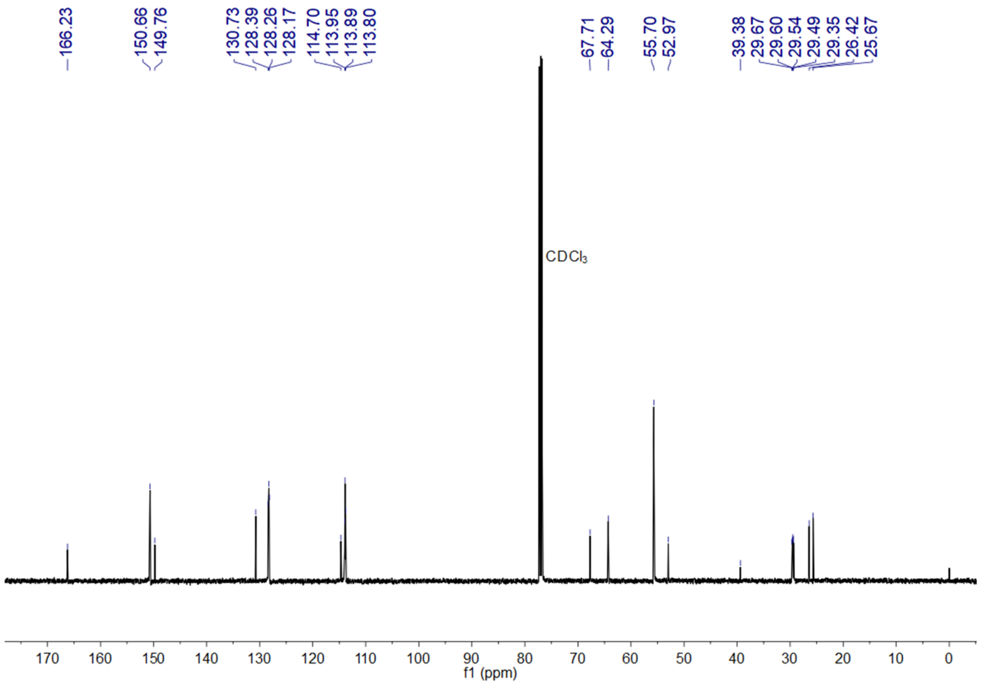

Figure S6. ^{13}C NMR spectrum (126 MHz, $CDCl_3$, 298K) of **AAP5A**.

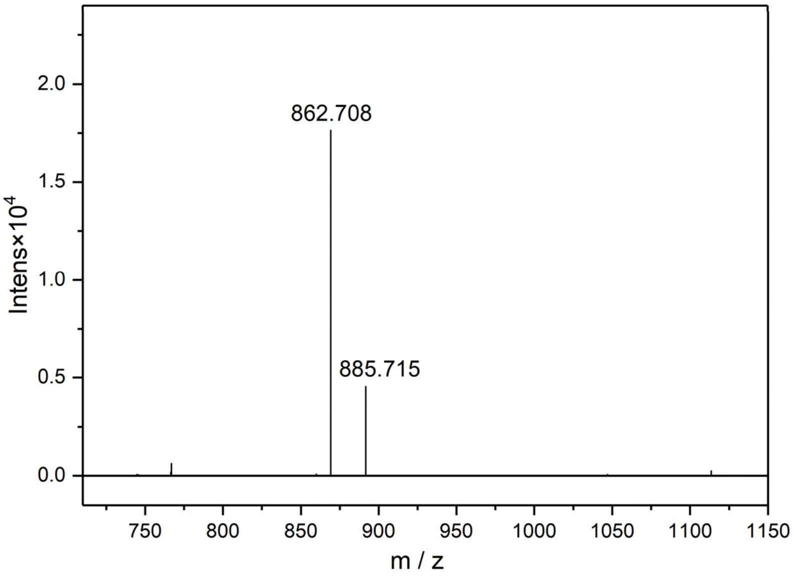

Figure S7. MALDI-TOF of **AAP5A**.

Synthesis of compound 3

1,6-dibromohexane (10.6g, 43mmol), K_2CO_3 (18.4g, 133mmol) and KI (3.32g, 20mmol) were dissolved in CH_3CN (100ml). After complete dissolution, acrylic acid

(2.4g, 33mmol) was added drop by drop. The reaction took place at 80°C under the protection of N_2 atmosphere for three days. After the reaction, the filtrate was cleaned and filtered by dichloromethane for several times. The filtrate was combined and concentrated. The crude product was further purified by silica gel column chromatography (eluent: petroleum ether/dichloromethane = 100:1, v/v). The yellow viscous liquid of 4.6g (yield 46%). The ^{1}H NMR spectrum of compound **3** is shown in **Figure S8**. ^{1}H NMR ($CDCl_3$, 500 MHz, 298K) δ (ppm): 6.40 (dd, J = 17.3, 1.4 Hz, 1H), 6.12 (dd, J = 17.4, 10.4 Hz, 1H), 5.82 (dd, J = 10.4, 1.5 Hz, 1H), 4.16 (t, J = 6.6 Hz, 2H), 3.41 (t, J = 6.8 Hz, 2H), 1.95 – 1.81 (m, 2H), 1.69 (dt, J = 14.2, 6.8 Hz, 2H), 1.55 – 1.33 (m, 4H). The ^{13}C NMR spectrum of compound **3** is shown in **Figure S9**. ^{13}C NMR (126 MHz, $CDCl_3$, 298K) δ (ppm): 166.29, 130.57, 128.54, 64.41, 33.69, 32.61, 28.45, 27.79, 25.17.

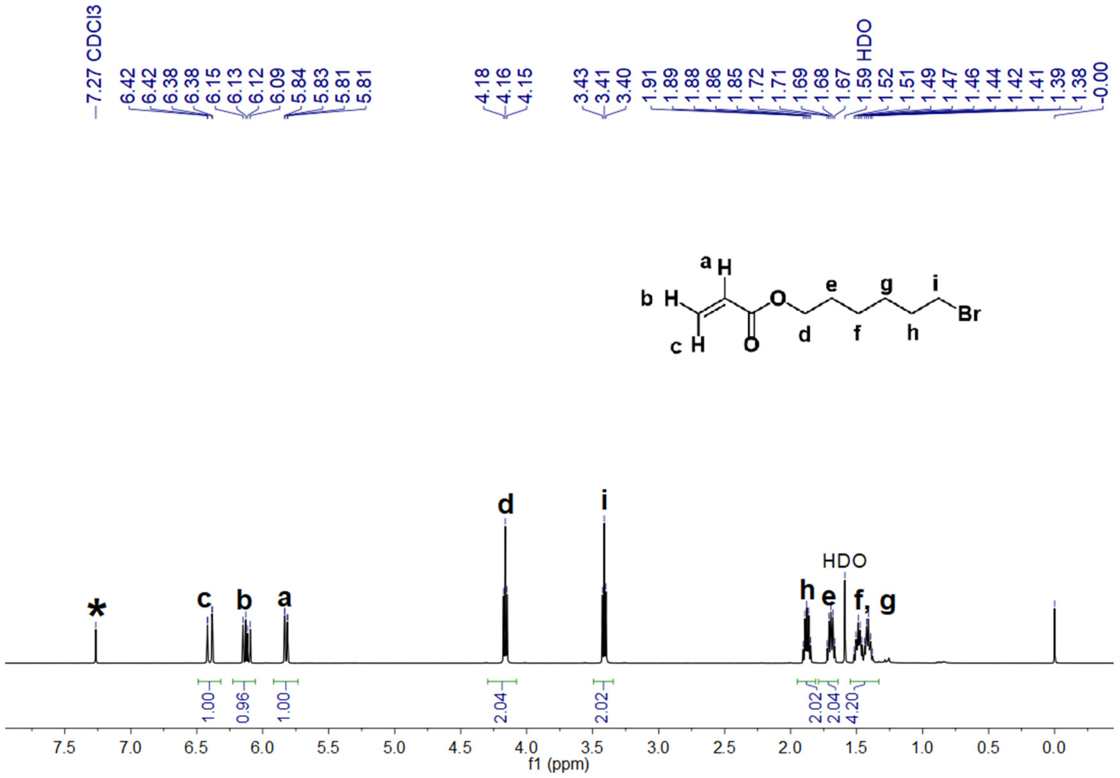

Figure S8. ^{1}H NMR spectrum (500 MHz, $CDCl_3$) of compound **3**.

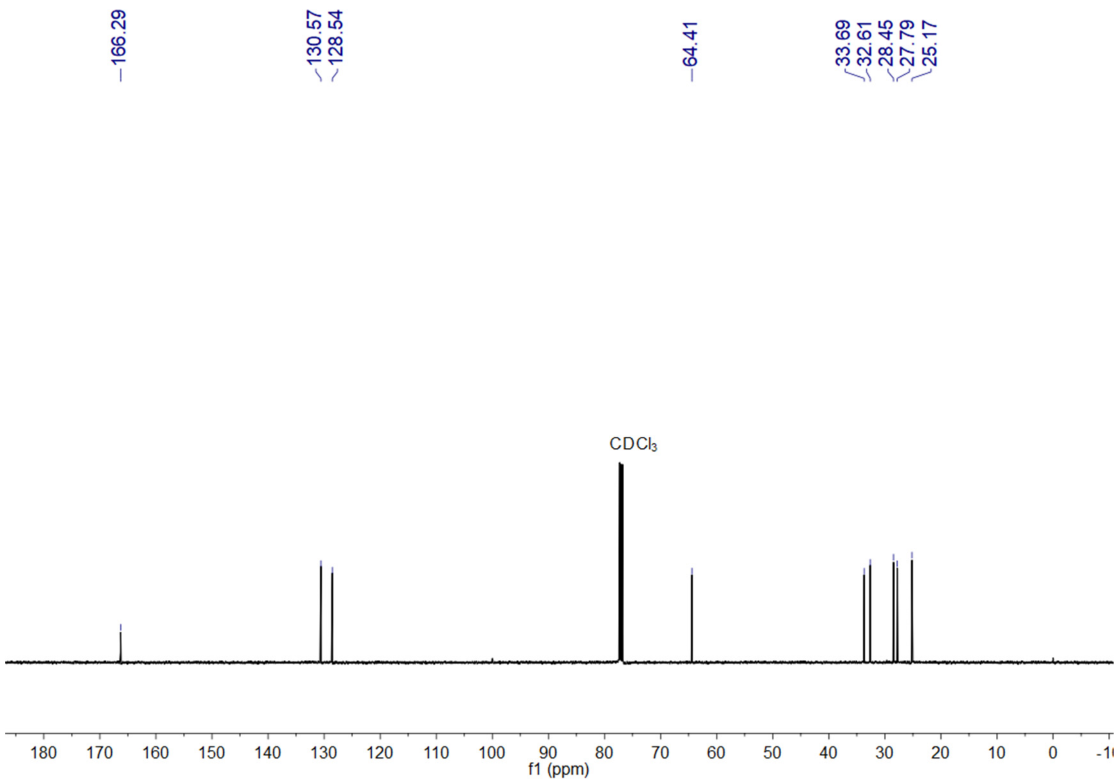

Figure S9. ^{13}C NMR spectrum (126 MHz, $CDCl_3$, 298K) of compound **3**.

Synthesis of pyridinium functionalized acrylate (G)

The compound **3** (1.0 g, 4 mmol) and pyridine (30 ml) were stirred at room temperature for three days, and the excess pyridine was removed by vacuum distillation. The resulting liquid was precipitated in ether to obtain a brown liquid of 0.92g (yield: 98%). The ^{1}H NMR spectrum of compound **G** is shown in **Figure S10**. ^{1}H NMR ($CDCl_3$, 500 MHz, 298K) δ (ppm): 9.54 (s, 2H), 8.54 (t, J = 7.9 Hz, 1H), 8.15 (t, J = 7.0 Hz, 2H), 6.38 (d, J = 17.2 Hz, 1H), 6.10 (dd, J = 17.4, 10.4 Hz, 1H), 5.82 (d, J = 10.4 Hz, 1H), 5.04 (t, J = 7.6 Hz, 2H), 4.12 (t, J = 6.6 Hz, 2H), 2.17 – 2.00 (m, 4H), 1.46 (s, 4H). The ^{13}C NMR spectrum of compound **G** is shown in **Figure S11**. ^{13}C NMR (126 MHz, $CDCl_3$, 298K) δ (ppm): 166.35, 145.32, 145.13, 130.82, 128.55, 128.46, 64.22, 61.86, 31.82, 28.29, 25.60, 25.35.

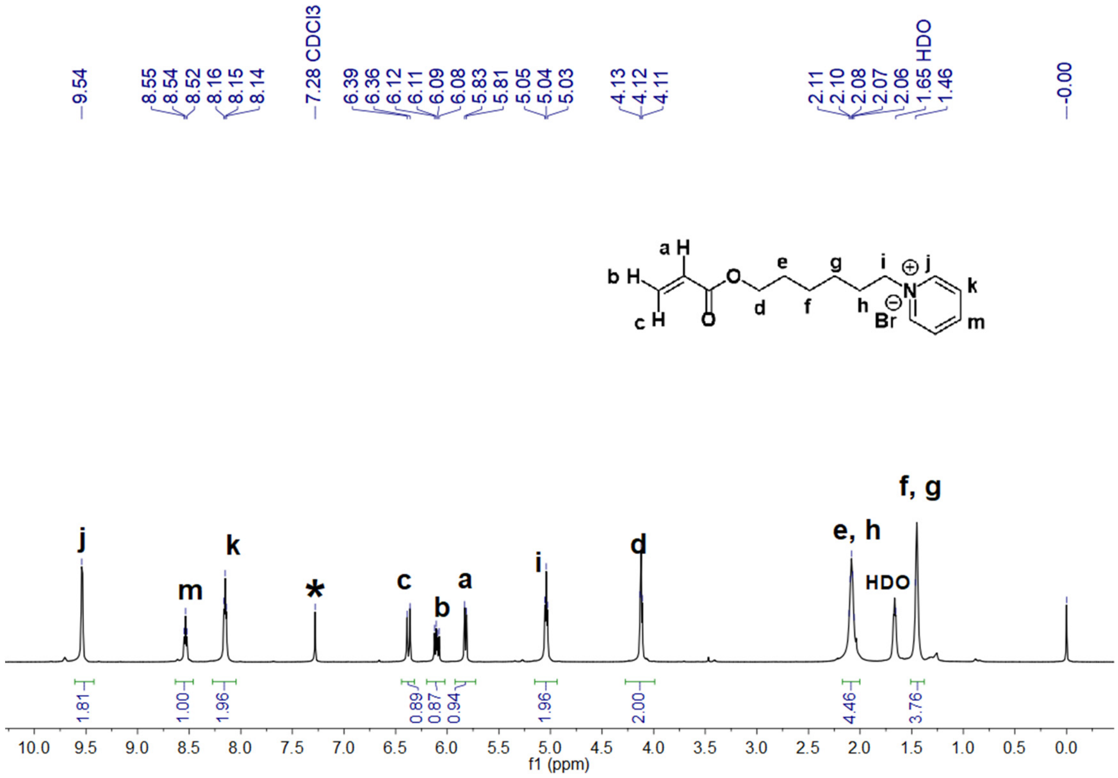

Figure S10. ^{1}H NMR spectrum (500 MHz, $CDCl_3$) of compound **G**.

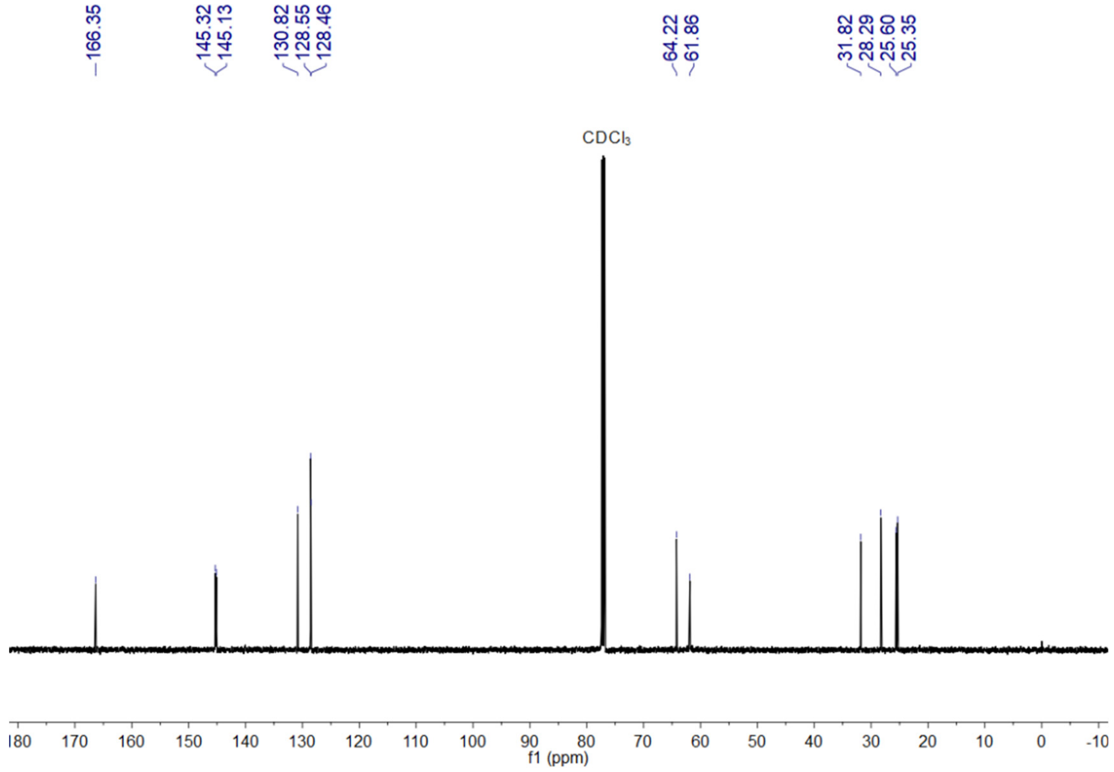

Figure S11. ^{13}C NMR spectrum (126 MHz, $CDCl_3$, 298K) of compound **G**.

2. Host-guest complexation studies of the supramolecular cross-linking molecule composed by AAP5A and G

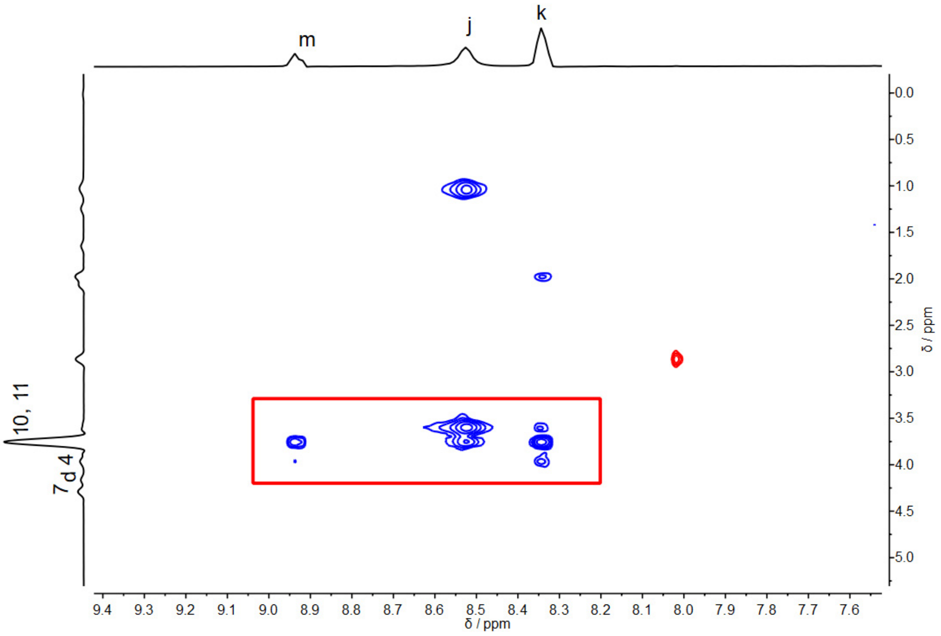

Figure S12. NOESY spectrum of a mixture of **AAP5A** (60.0 mM) and **G** (30.0 mM) (600 MHz, acetone-d_6, 298K).

NMR titration study of the complexation between AAP5A and G

The formation of supramolecular cross-linking molecule was studied by using **AAP5A** and **G** as templates. The complexation process was further studied by changing the concentration of **AAP5A** by fixing **G** concentration. For the ^{1}H NMR titration experiment, a solution of 4mM **G** was prepared first and mixed with **AAP5A** with a concentration of (a) 0.0 mM, (b) 0.8 mM, (c) 2 mM, (d) 3.2 mM, (e) 10 mM, (f) 20 mM, and (g) 40 mM.

First prepare solution (g): Dissolve 17.3 mg **AAP5A** (20 mmol) in the acetone-d_6 solution of 4 mM **G** (0.5mL);

(f) Add 0.5 mL solution of **G** (4 mM) to sample (g) and take out 0.5 mL.

(e) Add 0.5 mL solution of **G** (4 mM) to sample (f) and take out 0.5 mL.

(d) Add 1.06 mL solution of **G** (4 mM) to sample (e) and take out 0.5 mL.

(c) Add 0.30 mL solution of **G** (4 mM) to sample (d) and take out 0.5 mL.

(b) Add 0.75 mL solution of **G** (4 mM) to sample (c) and take out 0.5 mL.

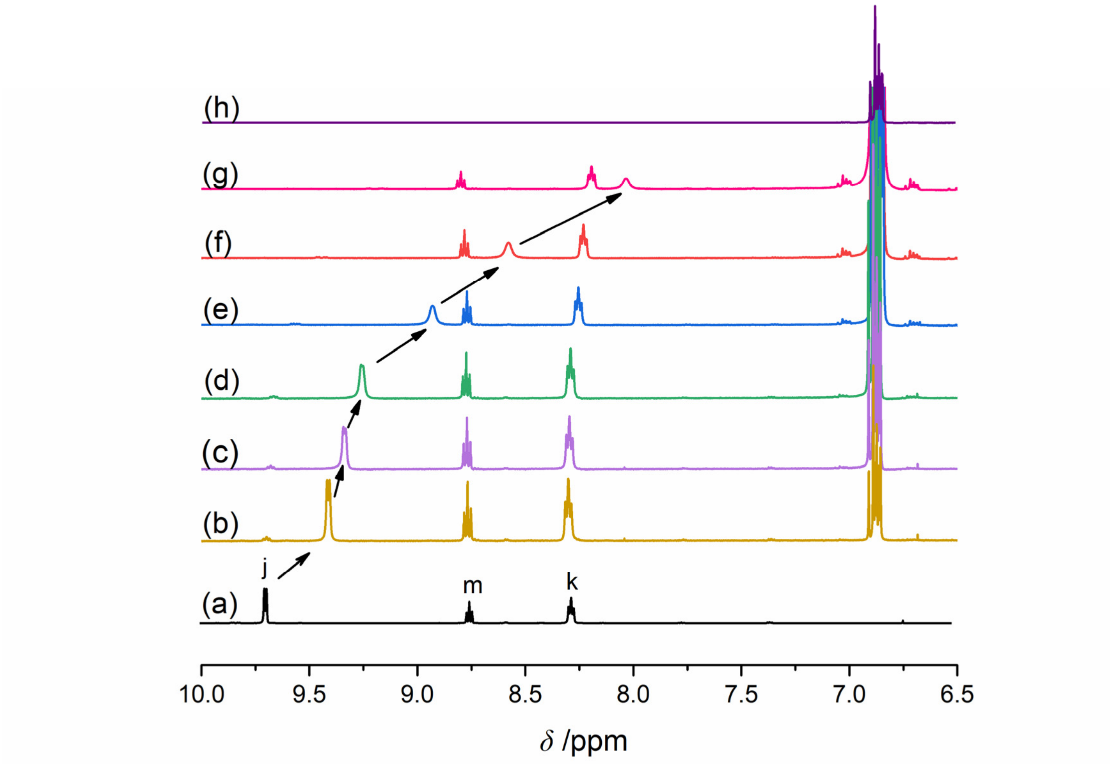

Figure S13. Partial ^{1}H NMR spectra (500 MHz, acetone-d_6, 298K) of **G** at a concentration of 4 mM upon addition of **AAP5A:** (a) 0.0 mM, (b) 0.8 mM, (c) 2 mM, (d) 3.2 mM, (e) 10 mM, (f) 20 mM, (g) 40 mM and (h) **AAP5A** at 8 mM.

Association constant determination for the supramolecular cross-linking molecules composed by AAP5A and G

The binding constant was obtained by the non-linear curve-fitting method. The association constant K_a between free **AAP5A** and **G** was calculated as (156± 41) mol^{-1}·L as shown in **Figure S15.** The binding constant was obtained by the non-linear curve-fitting method, using the equation: [S2,S3]

$\Delta\delta = (\delta_\infty / [G]_0) * (0.5 * [H]_0 + 0.5 * ([G]_0 + 1 / K_a) - (0.5 * ([H]_0 \wedge 2 + (2*[H]0 (1 / K_a - [G]_0)) + (1/ K_a + [G]_0) \wedge 2) \wedge 0.5))$

Where $\Delta\delta$ is the chemical shift change of pyridinium aromatic proton (H_j) owing to **G**, δ_∞ is the chemical shift change of these protons when the host is completely complexed, [**G**]$_0$ is the initial concentration of **G**, and $[H]_0$ is the varying concentrations of **AAP5A**.

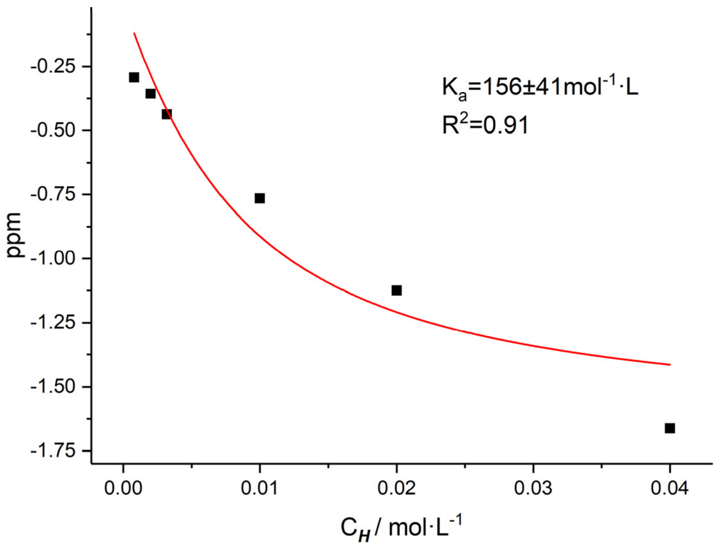

Figure S14. Fitting plot for the chemical shift changes of pyridinium aromatic proton(H_j) from **G** upon addition of **AAP5A**.

3. Preparation of the supramolecular polymeric material

Table S1. Feeding ratio of Covalent bond and supramolecular polymeric material.

**Sample**	**HGS (mM)**	**DEGDA (mM)**	**BA (mM)**	**TPO (mM)**	**Cross-linker content (%)**	**State**
1	-	1	3	2%	25%	gel*
2	-	1	5	2%	17%	gel*
3	-	1	10	2%	9%	gel*
4	-	1	15	2%	7%	gel*
5	-	1	20	2%	5%	gel*
6	1	-	3	2%	25%	--
7	1	-	5	2%	17%	--
8 (**SP**-1)	1	-	10	2%	9%	gel
9 (**SP**-2)	1	-	15	2%	7%	gel
10 (**SP**-3)	1	-	20	2%	5%	gel

* The mentioned sample cannot be tested for tensile properties; -- it cannot form a gel.

4. Characterizations of the supramolecular polymeric materials

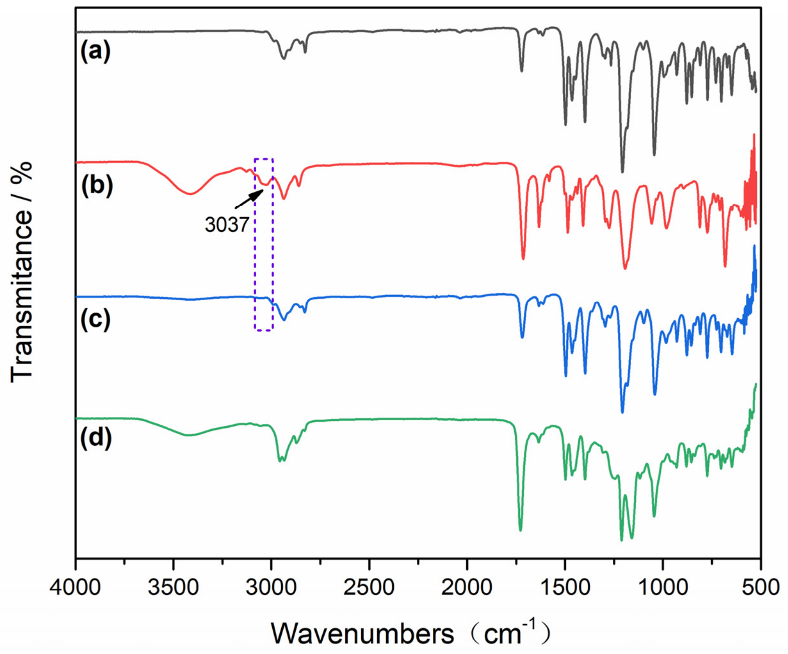

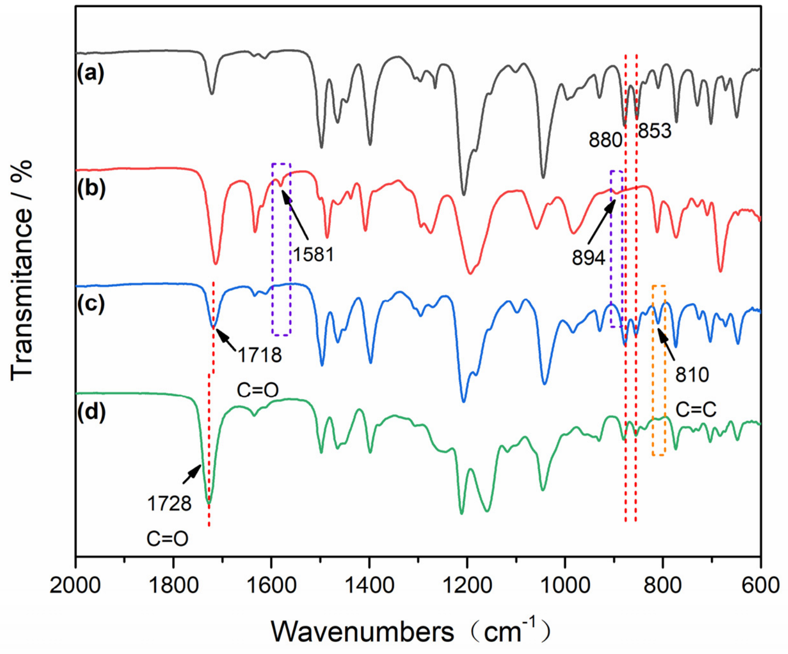

Figure S15. The full (upper) and partial enlarged (bottom) FT-IR spectra of **AAP5A** (a); **G** (b); **AAP5A⊃G** with a molar ratio of **AAP5A** to **G** at 2:1 (c) and **SP**-1 (d).

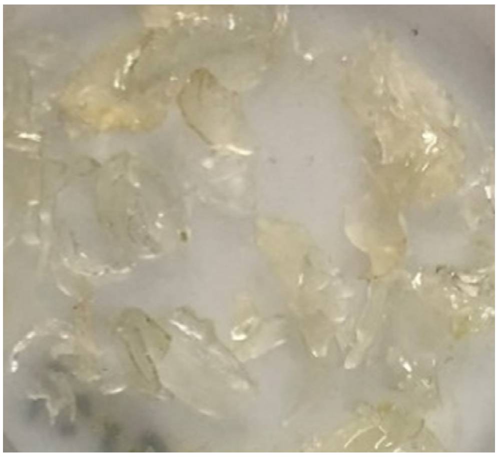

Figure S16. Picture of the covalent cross-linked polymeric material.

Table 2. Summary of mechanical properties.

**Sample**	**tensile strength * (MPa)**	**Strain at break* (%)**	**Toughness* ($MJ\cdot m^{-3}$)**
**SP**-1	3.39	400	12.24
**SP**-2	1.35	1300	16.11
**SP**-3	0.38	3040	8.47
pBA	0.11	1962	1.71

*** Strain rate = 30 $mm\cdot min^{-1}$**

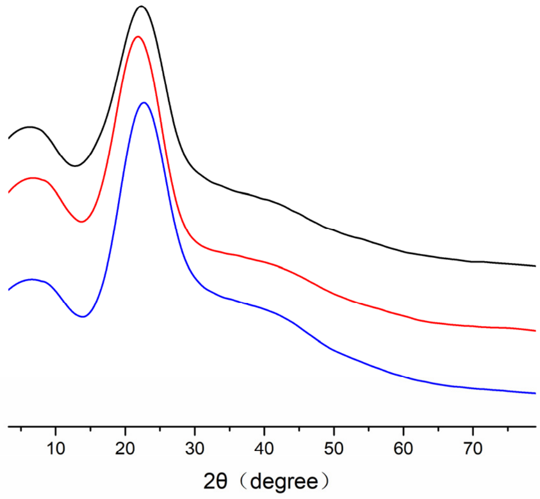

Figure S17. Wide angle X-ray scattering of **SP**-1 (black curve), **SP**-2 (red curve), and **SP**-3 (blue curve).

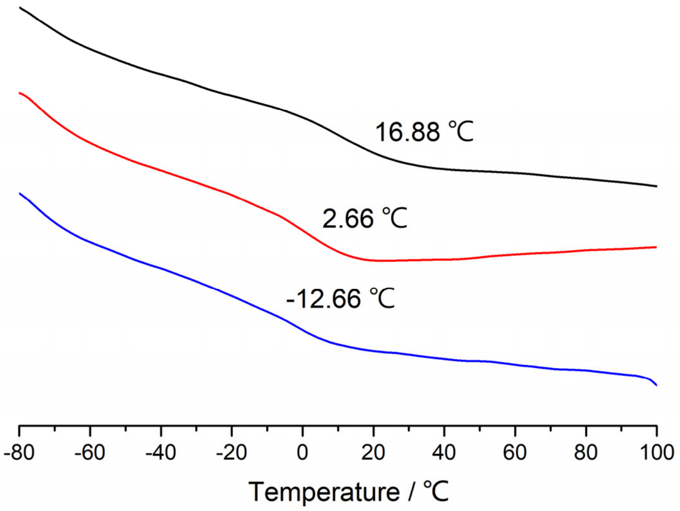

Figure S18. DSC curves of **SP**-1 (black curve), **SP**-2 (red curve), and **SP**-3 (blue curve) in the second heating process.

5. Self-healing property of the supramolecular polymeric materials

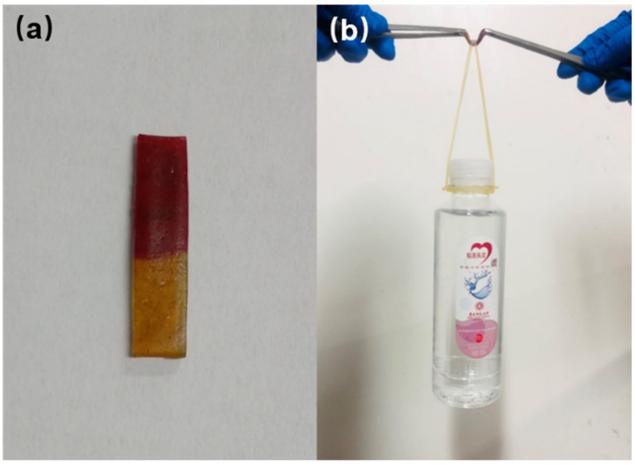

Figure S19. The optical image of the self-healing sample (one sample was stained with rhodamine B for better observation) and (b) the self-healing sample sustaining a load of 333 g.

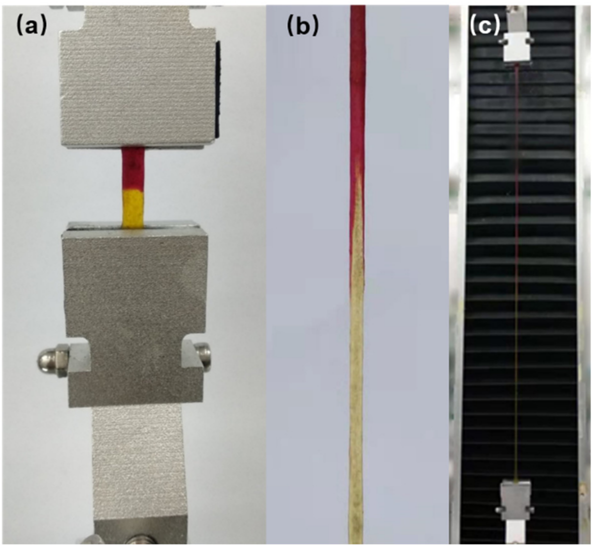

Figure S20. Photos of the original state of the self-healing sample before external force loading (a); the picture of the stretched detail of the healing site (b) and the healed sample under a large strain (c).

References

(S1) Wu, Y.; Li, H.; Yan, Y.; Shan, X.; Zhao, M.; Zhao, Q.; Liao, X.; Xie, M. Pillararene-containing polymers with tunable conductivity based on host–guest complexations. *ACS. Macro. Lett.* **2019**, *8,* 1588-1593.

(S2) Inoue, Y.; Yamamoto, K.; Wada, T.; Everitt, S.; Gao, X. M.; Hou, Z. J.; Tong, L. H.; Jiang, S. K.; Wu, H. M. Inclusion complexation of (cyclo)alkanes and (cyclo)alkanols with 6-O-modified cyclodextrins. *J. Chem. Soc.* **1998**, *2*, 1807-1816.

(S3) Peter R. A.; Roberto B.; Vincenzo B.; Martin B.; Maria T. G.; Douglas P.; Luca P.; Françisco M. R.; Mark V. R.; Neil S.; J. F. S.; Margherita V.; David J. W. Self-assembly, spectroscopic, and electrochemical properties of [n]rotaxanes. *J. Am. Chem. Soc.* **1996**, *118*, 4931-4951.
